# Median Duration of Hospital Stay after Early Removal of Foley’s Catheter among Patients Undergoing Transurethral Resection of Prostate: A Descriptive Cross-sectional Study

**DOI:** 10.31729/jnma.6384

**Published:** 2021-07-31

**Authors:** Pashupatinath Bhatta, Akash Raya, Umesh Kumar Yadav, Vijay Kumar, Sanjeev Shahi, Amit Singh

**Affiliations:** 1 Urology Unit, Department of Surgery, National Medical College, Birgunj, Nepal; 2 Department of Surgery, National Medical College, Birgunj, Nepal

**Keywords:** *benign prostatic hyperplasia*, *hospital stay*, *transurethral resection of prostate*, *urinary catheter*

## Abstract

**Introduction:**

Transurethral resection of the prostate requires a catheter in situ post-surgery. Early removal of a catheter can reduce the length of hospital stay reducing the healthcare cost. It can also reduce the risk of infection due to prolonged catheterization. Our aim was to determine the median duration of hospital stay after early foley's removal after transurethral resection of prostate among patients in a tertiary care hospital.

**Methods:**

A descriptive cross-sectional study was done in a tertiary care hospital from July 2019 to December 2020 and ethical clearance was obtained from the institutional review committee. Foley's catheter were removed on the first post-operative day, who met the criteria of catheter removal. Convenience sampling was done. After foley's removal patients were observed for spontaneous voiding. Patients with complications like hematuria, clot retention, urinary retention were recatherized. The data were expressed in mean with standard deviation, median with interquartile range and frequency and percentage as applicable using Statistical Package for the Social Sciences version 16.

**Results:**

Out of the 150 participants included in the study, the median duration of hospital stay after the early removal of foley's catheter was 3 days (interquartile range 2-4 days). A total of 20 (13.3%) patients underwent recatherization. Nine (6%) patients had to be recatheterized due to clot retention, and 11 (7.3%) were due to urinary retention.

**Conclusions:**

This study showed that the median duration of hospital stay after early removal of foley's catheter among patients undergoing transurethral resection of the prostate was similar to studies done in national/international settings.

## INTRODUCTION

Benign prostatic hyperplasia (BPH) is considered as a part of ageing process in male and is hormonally dependent on testosterone and dihydrotestosterone (DHT) production.^1^ There are various methods proposed for treatment of BPH, transurethral resection of prostate (TURP) remains gold standard.^2^ The increasing number of patients, long waiting list for TURP and lengthy hospital stay due to post-operative foley's catheterization is considered to be discomfort and cost disadvantage.^3^ This can be overcome by early removal of foley's catheter after TURP when appropriate and safe. As per various studies duration of hospitalization is 2-7 days. Among the total cost of TURP, 29-33% represent hospital stay charges.^4^

This study aimed to find out the median duration of hospital stay after early catheter removal on first postoperative day after TURP.

## METHODS

A descriptive cross-sectional study was done at National Medical College Department of Surgery, Urology Unit, Birgunj from July 2019 to December 2020. Ethical clearance was taken from the Institutional Review Committee (reference number: FNMC/439/075/76). The study was done among patients who underwent TURP and had early removal of foley's catheter. Patients undergoing TURP for BPH were included in the study. Bladder carcinoma, prostatic carcinoma, renal failure, cardiovascular diseases and other condition that require fluid restriction were excluded form the study. Digital rectal examination done in all cases to rule out nodularity. Convenience sampling was done and the sample size was calculated as,

n = Z^2^ × σ / e^2^

  = (1.96)^2^ × (0.5)^2^ × (0.08)^2^

  = 150

Where,

n = minimum required sample size
Z = 1.96 at 95% Confidence Interval (CI)
σ = standard deviation obtained from simiar study, 0.5^5^
e = margin of error, 8%

Therefore, the required sample size was 150 and the same number of participants were included. Patient were admitted one day before surgery. Positive urine culture were treated according to culture sensitivity report. All TURP performed under spinal anesthesia by a single urologist. TURP was performed according to standard technique by using glycine 1.5%. After completion of procedure prostate chips were evacuated by ellik evacuator and 22/24 3-way foley's catheter were inserted and bladder irrigation continued with normal saline, the catheter was placed on traction up to 6 hours. Age, the weight of resected prostate and re-operation, intraoperative complication, hospital stay were recorded.

The decision to remove the catheter was made by the urologist on next day at morning round. Criteria of catheter removal was normal vital sign, normal urine output, functioning irrigation channel, absence of clot, and adequate catheter effluent.

After removal of catheter patients were observed for spontaneous voiding. Recatherization was done in patients with severe hematuria, clot retention, urinary retention. Those patients who voided satisfactorily were discharged on next day with advise to follow up after one week with histopathological report.

The data was collected in data collection sheet and was entered in Microsoft Excel 2016. Data analysis was done using the Statistical Package for the Social Sciences (SPSS) version 16. The data were expressed in median with interquartile range, mean with standard deviation, and frequency with percentage where applicable.

## RESULTS

Out of the 1 50 paticipants of the study, the median duration of hospital stay was 3 (interquartile range 2-4) days with a minimum of two and maximum of seven days. In 130 (86.7%) cases, early foley's cathter removal was done with a minimum two and maximum five days. In 20 (13.3%) cases, recatherization was done in which the mean duration of hospital stay was 5 (interquartile range 4.75-6) days with minimum four and maximum seven days (Table 1).

**Table 1:** Duration of Hospital Stay.

Variables	n (%)	Median duration and interquartile range of hospital stay (days)	Minimum (days)	Maximum (days
Hospital Stay	150 (100)	3 (2-4)	2	7
Without Recatherization	130 (86.7)	3 (2-4)	2	5
With Recatherization	20 (13.3)	5 (4.75-6)	4	7

The minimum age of patient was 50 years and maximum age of patient on study is 90 years with mean age and standard deviation of 66.09±9.676. Maximum patients 53 (35%) were of the age group of 61-70 years (Table 2).

**Table 2:** Age-wise distribution of participants.

Age Group (In Years)	n (%)
41-50	6 (4)
51-60	42 (28)
61-70	53 (35)
71-80	42 (28)
>81	7 (5)
Total	150 (100)

The maximum time for surgery was 80 minutes and minimum time for surgery was 30 minutes with mean duration of surgery with standard deviation of 52.91±11.224 minutes. The maximum weight of resected prostate was 40 grams and minimum weight was 10 grams with mean weight of resected prostate was 20.37±6.381 grams (Table 3).

**Table 3:** Duration of surgery and weight of resected prostate.

Variables	Mean±S.D.	Maximum	Minimum
Duration of surgery (minutes)	52.91±11.224	80	30
Weight of resected Prostate (grams)	20.37±6.381	40	10

In total 150 patients, 20 (13.3%) patients underwent re-catherization. Catheter was removed on 5th day to 7th day after re-catherization, with mean and standard deviation of 4.4±1.086 (Table 4).

**Table 4:** Re-catherization.

Re-catherization	n (%)
Yes	20 (14)
No	130 (86)

**Figure 1:**
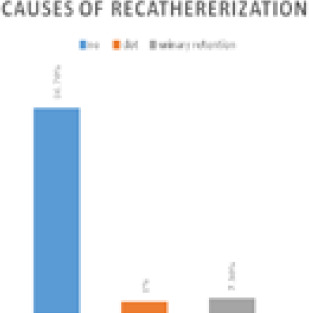
Bar diagram showing causes of recatherization.

Re-catherization was donein 9 patients (6%) due toclot and 11 (7.3%) (Figure 1).

## DISCUSSION

To reduce the length of hospital stay and cost-effectiveness of the procedure, there is tendency to decrease the length of in-dwelling foley's catheter after TURP. Early catheter removal is been practiced in number of places to determine the safety and efficacy of this approach.^3,6-11^ Study reported that in certain patient. Early catheter did not increase morbidity rather it reduces the cost and hospital stay.^3,6-11^

About one third of the total cost of TURP is represented by accommodation charges.^9^ The length of hospital stay for TURP decreased significantly between 1987 and 1995 from 10.6 to 6.1 days.^12^

The decreasing trend can be explained in basis of advancement of surgical technique and anaesthetic technique.

In this study, we have sucesfully removed the catheter on first POD in 130 (86%) cases ,where as 20 (14%) cases recatherized due to above mentioned complications. The median duration of hospital stay in early removal of catheter was 3 (interquartile range 2-4) days which was similar to study done by Chalise et al.^5^

Many clinical trials have emphasized on early catheter removal after TURP^6,7,11,13-18^ which helps in bed management, reduce cost and reduces the waiting list for TURP. Factors that influences the removal of catheter can be divided in three categories: Intrinsic patient factors, such as co-morbidities, urinary retention,hematuria,procedure specific factor such as resected prostate weight and intrinsic hospital factor and resourses.^11,12^

In one study it was found that duration of catheter removal related with resected prostatic weight,resected time and post-operative complication. we found that patient with no abnormalities, small prostatic size, less resection time and no peri operative complication were the candidate in whom we could remove catheter early and safely.

This study was done in a single institution with a limited sample size. Convenience sampling was done to enroll participants which might have introduced sampling error. Further multi-centric studies in a larger sample of patients which apply analytical study designs must be done in Nepal.

## CONCLUSIONS

This study showed that the median duration of hospital stay after early removal of foley's catheter among patients undergoing transuretheral reseaction of prostate was similar to studies done in national and international settings.
